# Atopic dermatitis

**DOI:** 10.1186/2045-7022-1-S1-S31

**Published:** 2011-08-12

**Authors:** Thomas Werfel

**Affiliations:** 1 Klinik für Dermatologie, Allergologie und Venerologie, Abteilung Immundermatologie und exp. Allergologiem Medizinische Hochschule Hannover, Hannover, Germany

## 

Among food allergens, cow's milk, hen's egg, wheat, soy, tree nuts and peanuts are most frequently responsible for exacerbation of atopic dermatitis (AD) in infancy. In older children, adolescents and adults pollen related food allergy should be taken into account. Different types of clinical reactions to food have been described in patients with atopic dermatitis: Early reactions occur within 120 minutes after the administration of the allergens. Late phase responses, manifesting as eczematous lesions, occur after 2-48 hours or some days. After oral food challenge, about 50% of children with AD who reacted to food showed both immediate and delayed reactions and 15% showed worsening of eczema only.The personal history is often not helpful predicting late reactions to food with a positive predictive value of only 30% as opposed to 80% for immediate reactions. Sensitizations to food can be identified by means of in vivo (skin prick tests, prick-prick tests) and in vitro tests (serum specific IgE). In addition, patch tests proved to be useful for studying delayed food-related skin responses. In vitro tests are valuable when skin prick tests, cannot be applied (e.g. dermographism) Moreover, in vitro specific IgE to food allergens give better quantitative data for the grade of sensitization which helps to estimate the probability of the risk of a clinical reaction (although precise decision points are not available) and it offers the opportunity to test single recombinant allergens. Atopy patch tests (APTs) which better reflect a T-cell mediated reaction are performed with self-made food material applied to the back with large test chambers for 48-72 hours. Food APT are not standardized for routine use but have demonstrated to improve the accuracy of skin testing in the diagnosis of allergy to cow's milk, egg, cereals, and peanuts in AD patients However, food challenge is not replaced by patch testing. The double-blind placebo-controlled food challenge (DBPCFC) is considered the gold standard for diagnosing food allergy. In AD the evaluation of delayed reactions after 24h or 48h by trained personal is mandatory as stated by a position paper of the EAACI.

